# Lifetime abuse and somatic symptoms among older women and men in Europe

**DOI:** 10.1371/journal.pone.0220741

**Published:** 2019-08-08

**Authors:** Bahareh Eslami, Mirko Di Rosa, Henrique Barros, Francisco Torres-Gonzalez, Mindaugas Stankunas, Elisabeth Ioannidi-Kapolou, Jutta Lindert, Joaquim J. F. Soares, Giovanni Lamura, Maria Gabriella Melchiorre

**Affiliations:** 1 Department of Occupational and Public Health Sciences, University of Gävle, Gävle, Sweden; 2 Division of Public Health Science, Department of Health Sciences, Mid Sweden University, Sundsvall, Sweden; 3 Laboratory of Geriatric Pharmacoepidemiology, National Institute of Health and Science on Aging, IRCCS INRCA, Ancona, Italy; 4 EPIUnit, Instituto de Saúde Pública da Universidade do Porto, Porto, Portugal; 5 Department of Psychiatry, Faculty of Medicine, University of Granada, Granada, Spain; 6 Department of Health Management, Lithuanian University of Health Sciences, Kaunas, Lithuania; 7 Health Service Management Department, School of Medicine, University of Griffith, Gold Coast, Queensland, Australia; 8 Department of Sociology, National School of Public Health, Athens, Greece; 9 Department of Public Health, University of Emden, Emden, Germany; 10 Women’s Studies Research Center, Brandeis University, Waltham, MA, United States of America; 11 Centre for Socio-Economic Research on Ageing, National Institute of Health and Science on Aging, IRCCS INRCA, Ancona, Italy; Nord University, NORWAY

## Abstract

**Background:**

Research suggests that survivors of interpersonal violence have an increasing experience of bodily symptoms. This study aims to scrutinise the association between lifetime abuse and somatic symptoms among older women and men, considering demographics/socio-economic, social support and health variables.

**Methods:**

A sample of 4,467 community-dwelling persons aged 60–84 years (57.3% women) living in seven European countries (Germany, Greece, Italy, Lithuania, Portugal, Spain, Sweden) was recruited for this cross-sectional study. Lifetime abuse (psychological, physical, sexual, financial and injury) was assessed on the basis of the UK study of elder abuse and the Conflict Tactics Scale-2, while somatic symptoms were assessed by the Giessen Complaint List short version.

**Results:**

Women reported somatic symptoms more frequently than men. Multiple regression analyses revealed that lifetime exposure to psychological abuse was associated with higher levels of somatic symptoms among both women and men, while experiencing lifetime sexual abuse was associated with somatic symptoms only among older women, after adjusting for other demographic and socio-economic variables. Country of residence, older age, and low socio-economic status were other independent factors contributing to a higher level of somatic symptoms.

**Conclusions:**

The positive association between the experience of abuse during lifetime and the reporting of higher levels of somatic symptoms, in particular among older women, seems to suggest that such complaints in later life might also be related to the experience of mistreatment and not only to ageing and related diseases. Violence prevention throughout lifetime could help to prevent somatic symptoms in later life.

## Introduction

In Europe, there is an increased concern about abuse and mistreatment against older persons. According to a recent systematic review and meta-analysis [1], the pooled prevalence rate for the overall elder abuse in the past year, against people living in community settings, is 15.7%, especially psychological (11.6%) and financial (4.2%) abuse. Other authors [2] have found that the prevalence of lifetime elder abuse across Europe is 34% for psychological abuse, 11.5% for physical abuse, 18.5% for financial abuse, and 5% for sexual abuse, with 4.3% of injuries.

Studies from some countries (e.g. Ireland, Israel, and the United Kingdom) have also reported that the larger number of victims of elder abuse concerns women, although different results have been found in other countries as men are more likely to experience abuse in later life, especially financial and emotional abuse [3]. The lifetime dimension also seems to occur often with regard to women. In particular, 50.6% of older women aged 60 years and over have reported psychological abuse, 6.2% physical abuse, and 3.5% sexual lifetime abuse [4]. Moreover, some studies have focused on the buffering effect of social support on elder abuse especially for women [5], also suggesting that low levels of perceived social support are related to older age and abuse across lifespan, particularly psychological abuse [6].

It is noteworthy that there is a shortage of research exploring the association between lifetime abuse and somatic symptoms in later life among both women and men within a cross-country perspective. The data available show that older persons often experience high rates of somatic symptoms [7], which are associated with poor health status, impaired social roles, and decreased quality of life [8]. Studies have also shown that women exceed men in reporting physical symptoms likely due to their biological assets, overwhelming social roles, higher physical information and awareness, and greater tendency to report symptoms [9]. Women’s health expenditure during lifetime is higher than that of men, and on the whole, over half of health care costs occur after the age of 65 [10]. Noteworthy, more than half of the population of older adults also suffer from multimorbidity [11], that is the presence of two or more concurrent long-term health conditions/diseases within a person [12, 13]. Therefore, the complex care needs of old multimorbid patients represent a great challenge to health systems and social services [14].

There is evidence that survivors of interpersonal trauma experience increased physical symptoms [9, 15]. A meta-analysis has shown that exposure to trauma increases the likelihood of somatic symptoms about 2.7 times [16]. It has also been reported that childhood and adolescent emotional/sexual abuse, including neglect, are correlated with the severity of somatic symptoms among middle-aged women [15]. Moreover, it has been suggested that although both female and male victims of sexual abuse, intimate partner violence and childhood trauma report a higher level of bodily symptoms, only women with adverse childhood experiences have a worse severity of somatic symptoms [9]. In particular, elder women experiencing abuse have increased odds of chronic pain, digestive and heart problems [17].

Most of the previous research (mentioned above) has thus focused on the impact of childhood maltreatment and intimate partner violence on somatic symptoms, especially among women. Childhood abuse seems to impact the life course of victims.

As societies age, it is crucial to understand the factors contributing to the physical and mental well-being of the elderly in order to improve policymaking and societal planning. Indeed, good health in later life helps the individual remain independent and autonomous, and have a better quality of life. Conversely, the occurrence of chronic diseases seems linked to inequalities in the physical and psychological quality of life among older adults [18]. In the light of these considerations, the current study aimed to examine the association between the experiences of lifetime abuse and somatic symptoms among older women and men living in Europe, considering socio-demographics, lifestyle, health variables (e.g. depressive symptoms) and social support variables. Based on previous few studies reporting on this topic, we hypothesised that somatic symptoms may be associated with the experience of abuse among older adults, both women and men across Europe.

## Materials and methods

### Study design and ethics statement

The present paper is based on data processed from the ABUEL survey (Elder Abuse: A multinational prevalence survey), a cross-sectional study conducted through face-to-face interviews or interviews/self-reports, in the following seven urban cities: Ancona (Italy), Athens (Greece), Granada (Spain), Kaunas (Lithuania), Stuttgart (Germany), Porto (Portugal) and Stockholm (Sweden). The final sample was randomly selected from the general population (census/registry-based). Written informed consents from participants were obtained prior to the data collection, and the study was approved by the national/university or regional ethics committees in each participating country, except for Greece where the QED Company (a member of the European Society for Opinion and Marketing Research, ESOMAR) provided ethical guidance.

The full names of the other six ethics committees/institutional review boards are as follows: Regional etisk kommittee vid Karolinska Institutet (Karolinska Institute, Regional Ethics Committee) in Sweden; Ethikkommission des Landes Baden-Wuerttemberg (Ethics Committee of the State of Baden-Wuerttemberg) in Germany; Comitato di Bioetica INRCA, Istituto Nazionale di Riposo e Cura per Anziani, Ancona (National Institute of Health and Science on Ageing, Bioethics Advisory Committee) in Italy; Kauno regioninio biomedicininiu tyrimu etikos komitetas (Kaunas Regional Research Ethics Committee) in Lithuania; Comité de Ética do Hospital de João, Porto (Ethics Committee of the John Hospital, Porto) in Portugal; Comité de Etica en Investigación de la Universidad de Granada (Research Ethics Committee, University of Granada) in Spain.

All survey materials (e.g. questionnaire) were culturally-adapted and translated, and followed a uniform protocol for administration and data treatment. In particular, in order to edit the questionnaire, a preliminary matrix of potential instruments was built (of which great part had already been translated into various languages), including related information e.g. validation for the elderly, sensitivity/specificity. Furthermore, specific guidelines regarding the translation process from English into the native language were followed: e.g. translation and back translation by qualified translators; review committee composed of two members, who were fluent in English and in the local language; and pilot testing of at least two interviews in each country for initial validity. Finally, after the piloting, some criticisms (e.g. misunderstood items/questions) were analyzed and adjusted by the review committee. Some cultural adjustments were also provided, by substituting any inappropriate/ambiguous items with others that better fitted the cultural target situation, while maintaining the general concept of the original items. Interviewers in each country were carefully instructed on how to manage the questionnaire, and on ethical behavior. Privacy, confidentiality, anonymity and voluntariness of data were carefully assured. Further details regarding the *Materials and Methods* section (study design, participants, and measures) have been published elsewhere [5, 6, 19].

### Participants

Community-dwelling older women and men were recruited for this study if they were: aged 60–84 years; living in own/rented housing or homes for elderly people; citizens or documented migrants (self-report); fluent in their native languages; and with no sensory or cognitive impairments (assessed by the Mini-Cog) [20] preventing them from completing study surveys. Recruitment of the participants and data collection occurred during the period January-July 2009. With a mean response rate of 45.2% across countries, the overall/final sample consisted of 4,467 older persons (57.3% women). The sample size was calculated based on the city’s population and the expected prevalence of abuse equal to 13%, as observed in previous surveys [21]. A total of 633 individuals in each country was established (with a 2.6% precision), but a maximum of 656 individuals was allowed in view of the infinite population assumption. This total was then customized according to the respective population aged 60–84 years.

### Measures

Lifetime abuse was assessed with 52 items based on the UK study of elder abuse [22] and the Conflict Tactics Scale-2 [23] assessing the exposure to psychological (11 items), physical (17 items), sexual (8 items), and financial abuse (9 items), in addition to abuse-related injury (7 items) after the age of 18, excluding childhood abuse. Lifetime abuse was defined as “at least 1 episode occurred during the past year and before.” For this purpose, we analyzed both current/last year and past abuse together (which, conversely, were collected separately in the questionnaire) in order to have an overall picture of all possible episodes of lifespan abuse experienced after the age of 18. Moreover, regarding the dichotomization of the measure of abuse, the responses to the items were processed as follows: “yes, abuse occurred during lifetime” as sum of (at least 1) episodes occurred once, twice, 3–5, 6–10, 11–20 or >20 times during the past year, and not occurred during the past year but before; “no, abuse did not occur during lifetime” when at least 1 episode did not occur/never occurred. The 52 items for collecting lifetime abuse in this study can be found as Supporting Information in this paper (S1 Text).

Somatic symptoms, that is the dependent variable of interest, were measured with the short 24-item version of the Giessen Complaint List (GBB) [24] comprising four domains (six questions each) of physical complaints including exhaustion (e.g. tiredness), gastrointestinal problems (e.g. nausea), musculoskeletal pain (e.g. pain in joints or limbs), and cardiac/heart distress (e.g. heavy, rapid or irregular heart-throbbing). Items were scored 0–4 (from “not affected” to “very much affected”), with a total somatic symptom score ranging from 0 to 96. Higher scores correspond to more somatic complaints. For this study, the focus was on the total scores (not on the domains). Cronbach’s Alpha for the entire scale across the total population/across countries was 0.92, and for the six complaints/individual sub-scales it was 0.82. Regarding internal consistency/construct validity, Pearson’s correlation coefficients (between each item questionnaire scores and total GBB score) ranged between 0.40–0.74, with 18 out of 24 items being over 0.50. The Giessen Complaint List (GBB 24) used in this study can be found as Supporting Information in this paper (S2 Text).

Multimorbidity, as the co-occurrence of two/more medical conditions in a person [12, 13] was measured in a yes/no format (i.e. more than one medical condition = yes; one or no medical condition = no). The medical conditions addressed were the following: Allergy, Asthma, Diabetes, Eye diseases (e.g. cataracts), Cardiovascular diseases (e.g. hypertension, stroke), Liver diseases (e.g. hepatitis), Stomach/bowel diseases (e.g. peptic ulcer), Lung diseases (e.g. chronic obstructive lung disease), and Cancer. Participants could choose the variable “other” and include further diseases suffered (e.g. arthritis).

Depressive and anxiety symptoms were assessed with the Hospital Anxiety and Depression Scale (HADS) [25], comprising 14 items (graded 0–3), with seven questions on depression (e.g. I feel as if I am slowed down) and seven on anxiety (e.g. I get sudden feelings of panic). Score ranges were from 0–21 for each scale, with higher scores suggesting worse anxiety and depressive symptoms. Moreover, for both dimensions, a score of 0–7 corresponded to no cases, 8–10 to possible cases, and 11–21 to probable cases. For this study, the focus was on the total score for anxiety and on the total score for depression, without considering respective cases cut-off.

Social support was assessed with the Multidimensional Scale of Perceived Social Support (MSPSS) [26] consisting of 12 questions (graded 1–7) divided into three domains, i.e. support from family, significant others and friends, with a total score ranging from 12–84. Higher scores indicated a greater social support perceived. This study focused on the total scores.

Socio-demographic variables were age (number of years), sex, relationship status (i.e. married/cohabiting, never married/widowed/divorced), educational achievement (i.e. low = informal/primary/similar; middle = high school/equivalent; high = university/similar), employment status (i.e. paid work, no work), main source of income/financial support (i.e. work, other income (partner’s), pension) and financial strain.

Lifestyle variables included: frequency (daily, weekly, monthly) of physical activities/ exercises (e.g. walking, swimming), and this dimension was scored positive when the participants did the activity at least 4 times a week; body Mass Index (BMI), based on self-reported height and weight (kg/m^2^); regular tobacco smoking, using a yes/no format; alcohol consumption, measured with a modified version of the Alcohol Use Disorders Identification Test (AUDIT) [27, 28].

### Data analysis

Analyses were performed with the PASW statistic package 24.0 (IBM/SPSS Inc., Chicago, IL) and STATA 15.1 (StataCorp, College Station, TX, USA). The Kolmogorov-Smirnov test was used to control the normality of distribution for the continuous variables. To examine group differences (i.e. women vs. men), we performed Student’s t-tests for continuous variables and chi-square tests for categorical variables. A multivariable linear regression model (based on the Ordinary Least Squares method), was used to examine the association between lifetime abuse and somatic symptoms adjusted per country of residence, age, sex, marital status, employment status, level of education, main source of income and financial strain. In order to highlight possible sex differences among each kind of lifetime abuse, interaction terms between these variables were included in the analysis. Therefore, we used two models: Model 1 as unadjusted model addressing the association between lifetime abuse/sex interaction and somatic symptoms; Model 2 as fully adjusted with the interaction term. To detect the multicollinearity of the regressors with the constant, Variance Inflation Factors (VIFs) were calculated. In the multivariable linear regression the data were expressed in B coefficients, p-values and R-square (R^2^). Missing values in Model 2 (n = 600) were excluded from the analysis. Model 3, including only the confounding variables, was developed in order to report the variance explained by the abuse variables over and above the confounding variables themselves. This value was calculated by subtracting the R^2^ of Model 3 from the R^2^ of Model 2. Finally, intercorrelation coefficients were calculated to analyse to what extent the types of abuse were intercorrelated. The statistical significance was set at p<0.05.

## Results

### Demographics, lifestyle/psychosocial variables, and lifetime abuse

As shown in Table 1, compared with men, women were slightly older, were less often in partnership and still working, with a lower educational level and a greater percentage of other income (e.g. social/sick leaves/benefits) than pension/work as the main source of income. Women also experienced greater financial strain, smoked cigarettes and drank alcohol less often, and were less physically active than men. Additionally, they referred higher mean scores in somatic complaints, anxiety and depressive symptoms (p<0.001), and social support (p = 0.002). Moreover, women reported multimorbidity and experienced in particular lifetime financial, sexual abuse and abuse-related injury more frequently than men (p<0.001).

**Table 1:** Demographic/socio-economic variables and lifetime abuse among women and men.

	Women (n = 2,559)		Men (n = 1,908)		
Variables	N (%)	Mean (SD)	N (%)	Mean (SD)	*p*
*Country*					*<0*.*001*
Germany	343 (13.4)		305 (16.0)		
Greece	356 (13.9)		287 (15.0)		
Italy	358 (14.0)		270 (14.2)		
Lithuania	405 (15.8)		225 (11.8)		
Portugal	400 (15.6)		256 (13.4)		
Spain	364 (14.2)		272 (14.3)		
Sweden	333 (13.0)		293 (15.4)		
*Age*^*a*^		70.50 (6.87)		69.90 (6.69)	*0*.*003*
*Married/cohabitant*	1366 (53.4)		1537 (80.6)		*<0*.*001*
*Education*					*<0*.*001*
Low	1004 (40.9)		613 (32.8)		
Middle	994 (40.5)		788 (42.1)		
High	458 (18.6)		470 (25.1)		
*Still working*	339 (14.3)		404 (21.2)		*<0*.*001*
*Main financial support*					*<0*.*001*
Work	241 (9.4)		301 (15.8)		
Pension	1470 (57.6)		1469 (77.1)		
Other income	843 (33.0)		136 (7.1)		
*Financial strain*	1751 (68.5)		1106 (58.1)		*<0*.*001*
*Smoking*	223 (8.7)		313 (16.4)		*<0*.*001*
*Alcohol consumption*	1400 (54.7)		1466 (76.8)		*<0*.*001*
*BMI*^*a*^ ^*b*^		26.64 (4.47)		26.72 (3.78)	0.532
*Physical activity*	1381 (54.0)		1177 (61.7)		*<0*.*001*
*Somatic symptoms*^*a*^		19.33 (15.96)		12.16 (12.03)	*<0*.*001*
*Depression*^*a*^^,^^*b*^		5.57 (4.24)		4.62 (3.76)	*<0*.*001*
*Anxiety*^*a*^^,^^*b*^		5.67 (4.23)		3.99 (3.55)	*<0*.*001*
*Social support*^*a*^^,^^*b*^		66.78 (15.23)		68.18 (13.82)	*0*.*002*
*Multimorbidity*					*<0*.*001*
2 or more diseases	1507 (58.9)		947 (49.6)		
*Lifetime abuse*					
Psychological	889 (34.7)		654 (34.3)		0.747
Physical	301 (11.8)		213 (11.2)		0.535
Injury	139 (5.4)		54 (2.8)		*<0*.*001*
Finanical	519 (20.3)		306 (16.0)		*<0*.*001*
Sexual	170 (6.6)		52 (2.7)		*<0*.*001*

n = Number; SD = Standard Deviation; *p* = p-value.

^a^ = range for continuous independent variables were as: Age: 60–84, BMI: 13.7–64.6, Somatic Symptoms: 0–85, Depression: 0–21, Anxiety: 0–21, Social support: 12–84.

^b^ = missing values for continuous independent variables were as: BMI: 97 for women, 47 for men; Depression: 32 for women, 24 for men; Anxiety: 35 for women, 20 for men; Social support: 66 for women, 43 for men.

### Lifetime abuse and somatic symptoms

As shown in Table 2, women exposed to psychological, physical, financial, sexual abuse and injury had higher significant scores on somatic symptoms than non-victims (p<0.001). Men exposed to psychological and financial abuse, and injury, scored significantly higher on somatic symptoms than their counterparts (respectively p<0.001, p<0.001, and p = 0.026).

**Table 2:** Lifetime abuse associated with somatic symptoms among women and men.

	Somatic symptoms			
	Women (n = 2,559)		Men (n = 1,908)	
Variables	Mean (SD)	*p*	Mean (SD)	*p*
*Psychological*		*<0*.*001*		*<0*.*001*
Yes	23.38 (17.49)		13.81 (12.63)	
No	17.18 (14.64)		11.29 (11.62)	
*Physical*		*<0*.*001*		0.698
Yes	27.91 (19.62)		12.98 (13.67)	
No	18.19 (15.05)		12.05 (11.81)	
*Injury*		*<0*.*001*		*0*.*026*
Yes	32.61 (21.71)		15.72 (12.75)	
No	18.51 (15.23)		15.05 (12.00)	
*Financial*		*<0*.*001*		*<0*.*001*
Yes	23.65 (16.87)		14.84 (12.80)	
No	18.23 (15.54)		11.64 (11.82)	
*Sexual*		*<0*.*001*		0.147
Yes	27.56 (18.79)		13.36 (10.07)	
No	18.75 (15.58)		12.12 (12.09)	

n = Number; SD = Standard Deviation; *p* = p-value

### Factors associated with somatic symptoms

As shown in Table 3, experiencing lifetime psychological and financial abuse were associated with higher levels of somatic symptoms among both women and men, whilst experiencing lifetime physical abuse and injury were associated with somatic symptoms only among older women (Model 1). After adjusting for other demographic and socio-economic variables, as potential confounding factors (Model 2), experiencing lifetime psychological abuse was still associated with higher levels of somatic symptoms among both women and men, whilst experiencing lifetime sexual abuse was associated with somatic symptoms only among older women.

**Table 3:** Demographic/socio-economic variables and lifetime abuse associated with somatic symptoms in multiple linear regression analyses among older women and men.

	Somatic symptoms					
	Model 1^d^		Model 2^e^		Model 3^f^	
	(n = 4,467)		(n = 3,867)		(n = 3,867)	
Variables	B	*p*	B	*p*	B	*p*
*Lifetime abuse* ^a^						
Psychological (ref. No)	2.20	*0*.*003*	1.46	*0*.*016*		
Psychological # Sex	1.42	0.141	0.70	0.384		
Physical (ref. No)	-1.35	0.256	-1.27	0.186		
Physical # Sex	4.26	*0*.*009*	2.29	0.096		
Injury (ref. No)	3.01	0.162	2.98	0.092		
Injury # Sex	5.33	*0*.*042*	1.72	0.438		
Financial (ref. No)	2.69	*0*.*003*	0.33	0.658		
Financial # Sex	0.91	0.429	0.61	0.526		
Sexual (ref. No)	-0.44	0.827	-1.29	0.433		
Sexual # Sex	3.97	0.091	4.82	*0*.*013*		
*Sex* (ref. Male)	5.27	*0*.*000*	1.94	*0*.*000*	2.93	*0*.*000*
*Age* ^b^			0.13	*0*.*000*	0.11	*0*.*000*
*Country* (ref. Germany) ^a^						
Greece			-1.82	*0*.*019*	-2.53	*0*.*001*
Italy			-2.70	*0*.*000*	-3.58	*0*.*000*
Lithuania			3.29	*0*.*000*	2.36	*0*.*001*
Portugal			2.60	*0*.*000*	2.83	*0*.*000*
Spain			0.91	0.268	0.75	0.360
Sweden			-2.52	*0*.*000*	-2.86	*0*.*000*
*Married/cohabitant* (ref. No) ^a^			-1.10	*0*.*008*	-1.19	*0*.*004*
*Education* (ref. Low) ^a^						
Middle			-1.93	*0*.*000*	-1.91	*0*.*000*
High			-2.62	*0*.*000*	-2.55	*0*.*000*
*Still working* (ref. No) ^a^			-0.19	0.804	-0.17	0.825
*Main financial support* (ref. Pension) ^a^						
Work			-0.08	0.923	-0.03	0.977
Other income			1.87	*0*.*000*	1.90	*0*.*000*
*Financial strain* (ref. No) ^a^			1.31	*0*.*001*	1.31	*0*.*001*
*Smoking* (ref. No) ^a^			0.71	0.197	0.66	0.236
*Alcohol consumption* (ref. No) ^a^			-0.20	0.640	0.04	0.929
*BMI* ^b^			0.20	*0*.*000*	0.23	*0*.*000*
*Physical activity* (ref. No) ^a^			-1.08	*0*.*005*	-1.05	*0*.*007*
*Depression* ^b^			0.63	*0*.*000*	0.66	*0*.*000*
*Anxiet*y ^b^			0.89	*0*.*000*	0.93	*0*.*000*
*Social support* ^b^			-0.02	0.203	-0.03	*0*.*022*
*Multimorbidity* ^c^ (ref. No) ^a^			6.37	*0*.*000*	6.59	*0*.*000*
*Constant*	11.05	*0*.*000*	-9.12	*0*.*003*	-7.25	*0*.*017*

^a^ = Categorical Variables

^b^ = Continuous Variables

^c^ = Multi-morbidity: to have 2 or more diseases

B = B coefficients; *p* = p-value; # = interaction

^d^ = R^2^ Model 1: 0.1097

^e^ = R^2^ Model 2: 0.4425

^f =^ R^2^ Model 3: 0.4301.

Moreover, as shown in Table 3, in Model 2 somatic symptoms were positively associated with coming from Lithuania or Portugal, being older age, female, having the main source of income other than work/pension, financial strain, higher BMI, elevated anxiety and depressive symptoms and multiple morbidities. Coming from Greece, Italy or Sweden, living in partnership, middle/high educational level and regular physical activity seem to act as “protective factors.” Model 2 could explain 44% of the variance in somatic symptoms. The abuse variables in Model 1 could explain 11% of the somatic symptom variance. However, by developing Model 3 we found a low value of 0.0124 (1%) as a variance explained by the abuse variables, over and above the confounding variables (abuse effect size). This value was calculated as difference between R^2^ Model 2 (0.4425) and R^2^ Model 3 (0.4301). As for the validity of the analyses, for Model 1 mean VIF was 3.53, while for Model 2 it was 2.26, confirming that no collinearity issue existed. The coefficients of the intercorrelation matrix, between 0.10–0.35 (apart from physical abuse and injuries, which was around 0.50), also confirmed the absence of multicollinearity (Table 4).

**Table 4:** Types of lifetime abuse: Coefficients of the intercorrelation matrix ^a^.

Variables	Psychological	Physical	Injuries	Financial	Sexual
Psychological	1.00				
Physical	0.35	1.00			
Injuries	0.22	0.52	1.00		
Financial	0.17	0.16	0.11	1.00	
Sexual	0.21	0.25	0.20	0.15	1.00

^a^ = Point-biserial correlation coefficients

## Discussion

The overall findings presented in this paper seem to suggest an association between the experience of lifetime elder abuse and somatic complaints, although the abuse effect size is small (only 1%). Our findings are in accordance with previous studies, which highlight on the whole that negative physical/emotional events negatively impact health and well-being [29], although in particular the association of psychological abuse with somatic complaints emerged smaller (as β-values of multiple linear regression) when compared with that of other dimensions (e.g. mental health) and again somatic pains [30].

In particular, our results highlighted that older women, more frequently than men, reported such complaints in a general population sample from seven European countries. Moreover, the multiple regression analyses indicated that lifetime exposure to sexual abuse was associated with a higher level of somatic symptoms among women, whereas the exposure to psychological abuse during the life course was associated with a higher level of somatic symptoms among both men and women. Although multimorbidity affects more than half of the population of older adults [11], the lifetime abuse association with somatic symptoms was independent of multimorbidity in our sample. It has been reported that multimorbidity does not differ between abused and non-abused older persons [31]. Our findings indicate that lifetime abuse could result in somatic symptoms in old age, independent of a particular disease. However according to some authors [32], particularly stressful life events (e.g. violence in the home and sexual abuse) represent a dimension mediating the association multimorbidity-loneliness, especially for adults aged 65 and over. More research is needed to further examine the link between elder abuse and health outcomes. It is also important to distinguish health consequences of elder abuse from natural processes related to aging and diseases.

In line with our results, it has been highlighted that women with a lifetime history of sexual abuse by their intimate partners experience high levels of disabling chronic pain [33]. A painful event, such as intimate partner violence, may indeed have long-term negative effects on health beyond the time of the violence itself. It is also possible that exposure to violence increases the awareness of somatic symptoms [34]. Other authors have shown a significant association between experiencing childhood and adolescent emotional and sexual abuse and severity of somatic symptoms [15], and have also reported that, for older men, the probability of being abused increases along with the increase of somatic and anxiety symptoms [35]. In particular, psychological abuse emerged as related to somatic symptoms; however, also other factors (e.g. depression, anxiety, social support) played an important role in this context [30].

It has also been shown that body regulatory systems (e.g. neuroendocrine), which are responsible for adaptation to internal and external stressors, may malfunction due to the superimposition of excess stressful events [36]. Therefore, one may argue that the cumulative effect of exposure to lifetime abuse could lead to allostatic overload. On the other hand, sex differences observed in the type of abuse and somatic symptoms may pertain to differences in coping strategies, cognitive appraisal and reappraisal of the stressors, which may exist among women and men. Future studies aimed at exploring these factors may help to understand the mechanism behind these findings.

We furthermore observed that the association between exposure to lifetime abuse and somatic symptoms was independent of current anxiety and depressive symptoms among older women and men. Various studies have highlighted the co-morbidity between somatic symptoms, anxiety and depression [37, 38]. In particular, some researchers [8] have suggested that somatic symptoms could be the expression of certain psychological problems such as depression, while others [9] have shown that there is a pathway from interpersonal trauma to somatic symptoms through depression. In this regard, they have proposed to decrease depressive symptoms among survivors of abuse to mitigate the trauma’s effect on somatic symptoms.

In line with some previous researches [7, 39, 40], we also found that older age, socio-economic disadvantages, and geographical location were positively associated with somatic symptoms in this sample. It is noteworthy that the country of residence was a risk factor for some countries (e.g. Lithuania) and a sort of “protective factor” for others (e.g. Sweden). Some previous findings have also shown that, for instance, older persons from Lithuania (and Portugal) considered their health to be worse than that of older persons from Sweden (and Italy) [39]. This may pertain to the differences in the availability and accessibility of welfare systems as well as the presence of social and health equality/equity in those countries. As mentioned, the standard of living of older people differs across these countries [41]. However, the country differences in reporting somatic symptoms seem to be more related to country “characteristics” (e.g. availability of good health care services) than to country “cultural patterns” [30].

### Limitations

This study has some limitations. First of all, it is to be acknowledged that the variance explained by the abuse variables, over and above the confounding variables (abuse effect size) is only 1%. Moreover, the translated/adapted questionnaires were not tested psychometrically for cross-cultural “measurement equivalence” (ME) [42], as comparability of measured attributes across different populations. Therefore, although detailed guidelines for the translation process were followed (as specified in the “Study design” section), the pooled results obtained should be interpreted with caution, due to the possible inclusion of terms that might have a culture/country specific feeling/interpretation/expression. This could potentially affect a valid comparison of cross-cultural data for the interpretation of the results, and limit the conclusions drawn from the study, also with regard to policy implications as suggestion/provision of possible adequate intervention policies for detection/prevention of elder abuse. This consideration particularly affects such sensitive issue/phenomenon, due to country/cultural/social norms influencing behaviours, attitudes and perceptions in this respect. Also, the data concerned only large urban centres in seven European countries. Older adults with cognitive impairments were excluded from the study, thus the generalizability of the findings should be read with caution. The cross-sectional design prevented to establish causal links between somatic symptoms and its covariates, given that this would require another design type (e.g. longitudinal study with repeated measures). Therefore, somatic symptoms may be both effects of and risk factors for elder abuse. Elder abuse may in turn be both consequence of and risk factor for somatic symptoms. Moreover, the study relied on the participants’ self-reports and did not use objective measures, with a possible general and/or differential misclassification and bias. For instance, the presence of pain complaints was not objectively confirmed, and more severely ill persons may have refused to be interviewed. However, subjective measures of psychosocial well-being are appropriate. Furthermore, participants were asked about exposure to abuse after the age of 18, and this may also have resulted in recall bias. Finally, this study had a relatively low response rate (45.2%) across countries, which may have resulted in an under-reporting bias, but this seems also related to general population-based studies addressing sensitive issues such as abuse. Despite these limitations, our study, with a relatively large sample size, provided cross-national data on various aspects of elder abuse, presented a workable definition of abuse and used, when possible, validated instruments/measures to assess the phenomenon. However, in order to carry out a cross-country and exploratory/pilot study on the prevalence of elder abuse, we were interested in any single episode of abuse (at least 1) during life-course (and last year). And this differently from other studies, which conversely followed substantive threshold criteria thus collecting, for instance, a case of psychological abuse only when at least 10 or more episodes had occurred [3]. Our larger operational definition of lifetime abuse might thus have impacted on a (greater) prevalence rate in our study. This may have further impacted on the association between lifetime abuse and levels of somatic symptoms, with larger rates of victims (according to our definition) experiencing somatic symptoms.

## Conclusions

Despite the limitations of the study, especially the one regarding the definition/measurement of lifetime elder abuse (at least 1 episode during life-course), in addition to the small abuse effect size, the current study confirmed on the whole previous findings and may have provided new insights into the experience of somatic complaints in connection to lifetime abuse. Indeed, our study suggests that older women who experienced psychological and sexual abuse during their life course, as well as older men who experienced psychological violence, also reported higher levels of somatic symptoms compared to those who had never been exposed to abuse. The observed association was independent of multimorbidity and mental ill-health. Moreover, our study found that the country of residence and socio-economic disadvantages can play a role in subjective somatic symptoms in later life. Our findings, regarding the positive association between lifetime abuse and higher levels of somatic symptoms, in particular among older women, seem to suggest that violence prevention throughout life course can contribute to contrast somatic symptoms in later life. The implication of the present study seems indeed that health care providers/planners/policymakers should acknowledge that somatic symptoms among older people, in particular women with low socio-economic status, might also be associated to the experience of violence during life course, and not only to diseases which are usually linked to the ageing process. Violence prevention during lifetime could thus be of some help to prevent somatic complaints in later life. More generally, with regard to healthcare workers, there is a crucial need to improve their perception/knowledge and concern of possible cases of elder abuse and related risk factors and health consequences, in order to increase their “willingness to deal with the problem” [43].

## Disclaimer

The authors have adapted some brief parts of the text from their own previous publication concerning the same main ABUEL Study, with appropriate attribution. The adapted text refers to some general socio-demographic information of the sample population, ‘Materials and Methods’ (as well established protocols), and ‘Limitations’. The previous publication is the following: Melchiorre MG, Chiatti C, Lamura G, Torres-Gonzales F, Stankunas M. Lindert J, et al. Social support, socio-economic status, health and abuse among older people in seven European Countries. *PLoS ONE* 2013;8:e54856, doi: 10.1371/journal.pone.0054856.

## Supporting information

S1 Text 52 Items for lifetime abuse. (DOC)

S2 Text Giessen complaint list (GBB-24). (DOCX)
