# Supplementary material for: Lifetime abuse and somatic symptoms among older women and men in Europe
Source: PLoS One. 2019 Aug 8;14(8):e0220741. doi: 10.1371/journal.pone.0220741 (PMC6687146; doi:10.1371/journal.pone.0220741)
Supplement: S1 Text — (DOC) [file pone.0220741.s001.doc]

**ABUEL Study**

**S1 Text. 52 Items Lifetime abuse**

**For each type of abuse:**

*How many times the interviewed has been exposed to each of the events indicated below:*

1 = Once in the past year 5 = 11-20 times in the past year

2 = Twice in the past year 6 = More than 20 times in the past year

3 = 3-5 times in the past year 7 = Not in the past year, but happened before

4 = 6-10 times in the past year 8 = This has never happened

**Psychological**

Someone has/some people have……

| 1. Insulted you or sworn at you |
| --- |
| 2. Threatened you (*e.g. putting you in a nursing home, brea-king things that you care about*) |
| 3. Undermined or belittled what you do |
| 4. Excluded you or repeatedly ignored you |
| 5. Threatened to harm others that you care about (*e.g. pets, relatives*) |
| 6. Prevented you from seeing others that you care about |
| 7. Shouted or yelled at you |
| 8. Did something to spite you |
| 9. Called you fat, ugly or other names |
| 10. Destroyed something that belonged to you |
| 11. Threatened to hit or throw something at you |

**PHYSICAL**

Someone has/some people have……

| 1. Slapped you |
| --- |
| 2. Grabbed you |
| 3. Kicked you |
| 4. Pushed or shoved you |
| 5. Burned or scalded you on purpose |
| 6. Choked you |
| 7. Threw something at you that hurt |
| 8. Twisted your arm or hair |
| 9. Used a knife, a gun or other weapon on you |
| 10. Punched or hit you with something that could hurt |
| 11. Slammed you against a wall, door or a piece of furniture |
| 12. Beat you up |
| 13. Tied you down |
| 14. Restrained you in any other way |
| 15. Locked you in your room |
| 16. Gave you drugs or too much medicine to control you/ make you docile |
| 17. Threatened you with knife, a gun or other weapon |

**PHYSICAL INJURY**

| 1. You had a sprain, bruise or small cut from being hit etc. |
| --- |
| 2. You passed out from being hit on the head |
| 3. You went to the doctor from being hit etc. |
| 4. You needed to go to the doctor from being hit etc, but did not |
| 5. You had a broken bone from being hit etc. |
| 6. You felt a physical pain that still hurt the next day from being hit etc. |
| 7. Other injury, please specify |

**FINANCIAL**

Someone has/some people have……

| 1. Made you give him/them your money, possessions or property against your will |
| --- |
| 2. Tried to make you give money, possessions or property |
| 3. Tried to use fraud to take money, possessions or property from you |
| 4. Tried to take or keep power of attorney against your will |
| 5. Attempted to steal money, possessions or property from you |
| 6. Stolen money, possessions or property from you |
| 7. Used fraud to take your money, possessions or property from you |
| 8. Took or kept power of attorney over you against your will |
| 9. Did something else to take your money, possessions or property from you |

**SEXUAL**

Someone has/some people have……

| 1. Talked to you in a sexual way |
| --- |
| 2. Touched you in a sexual way against your will |
| 3. Tried to touch you in a sexual way against your will |
| 4. Made you watch pornography against your will |
| 5. Tried to make you watch pornography against your will |
| 6. Had sexual intercourse with you against your will |
| 7. Tried to have sexual intercourse with you against your will |
| 8. Other sexual molesting behaviours |
