# Supplementary material for: Lifetime abuse and somatic symptoms among older women and men in Europe
Source: PLoS One. 2019 Aug 8;14(8):e0220741. doi: 10.1371/journal.pone.0220741 (PMC6687146; doi:10.1371/journal.pone.0220741)
Supplement: S2 Text — (DOCX) [file pone.0220741.s002.docx]

**ABUEL Study**

**S2 Text. Giessen Complaint List (GBB-24)**

|  |  | Not at all  (affected)  ▼**_1_** | Slightly  ▼**_2_** | Some-what  ▼**_3_** | Consider-ably  ▼**_4_** | Very much  (affected)  ▼**_5_** |
| --- | --- | --- | --- | --- | --- | --- |
| 1) | Physical weakness | _1_ | _2_ | _3_ | _4_ | _5_ |
| 2) | Heavy, rapid or irregular heart-throbbing | _1_ | _2_ | _3_ | _4_ | _5_ |
| 3) | Pressure or heaviness in the stomach | _1_ | _2_ | _3_ | _4_ | _5_ |
| 4) | Excessive need for sleep | _1_ | _2_ | _3_ | _4_ | _5_ |
| 5) | Pains in joints or limbs | _1_ | _2_ | _3_ | _4_ | _5_ |
| 6) | Dizziness | _1_ | _2_ | _3_ | _4_ | _5_ |
| 7) | Backache | _1_ | _2_ | _3_ | _4_ | _5_ |
| 8) | Pains in neck or shoulders | _1_ | _2_ | _3_ | _4_ | _5_ |
| 9) | Vomiting | _1_ | _2_ | _3_ | _4_ | _5_ |
| 10) | Nausea | _1_ | _2_ | _3_ | _4_ | _5_ |
| 11) | Sensation of tightness, choking or lumpiness in the throat | _1_ | _2_ | _3_ | _4_ | _5_ |
| 12) | Belching | _1_ | _2_ | _3_ | _4_ | _5_ |
| 13) | Heartburn | _1_ | _2_ | _3_ | _4_ | _5_ |
| 14) | Headaches | _1_ | _2_ | _3_ | _4_ | _5_ |
| 15) | Tendency to rapid exhaustion | _1_ | _2_ | _3_ | _4_ | _5_ |
| 16) | Tiredness | _1_ | _2_ | _3_ | _4_ | _5_ |
| 17) | Feeling numb or benumbed | _1_ | _2_ | _3_ | _4_ | _5_ |
| 18) | Heaviness or tiredness in the legs | _1_ | _2_ | _3_ | _4_ | _5_ |
| 19) | Weariness | _1_ | _2_ | _3_ | _4_ | _5_ |
| 20) | Twinges, pains or aching in the chest | _1_ | _2_ | _3_ | _4_ | _5_ |
| 21) | Stomach-aches | _1_ | _2_ | _3_ | _4_ | _5_ |
| 22) | Attacks of breathlessness | _1_ | _2_ | _3_ | _4_ | _5_ |
| 23) | Head-pressure | _1_ | _2_ | _3_ | _4_ | _5_ |
| 24) | Sudden bouts of heart-trouble | _1_ | _2_ | _3_ | _4_ | _5_ |

### Sub-scale "**Exhaustion tendency**" (Erschöpfung) items: 1, 4, 15, 16, 17, 19.

### Sub-scale "**Stomach discomfort or gastric/gastrointestinal symptoms**" (Magenbeschwerden) items: 3, 9, 10, 12, 13, 21.

### Sub-scale "**Body/musculoskeletal aches or pain in the limbs**" (Gliederschmerzen) items: 5, 7, 8, 14, 18, 23.

### Sub-scale "**Cardiac or Heart complaints/distress**" (Herzbeschwerden) items: 2, 6, 11, 20, 22, 24.
